# Cavernous Sinus Endodermal Cyst: A Report of a Rare Case

**DOI:** 10.7759/cureus.85497

**Published:** 2025-06-07

**Authors:** Shintaro Arai, Tohru Mizutani, Katusyoshi Shimizu, Kouzou Murakami, Yoichi Morofuji

**Affiliations:** 1 Department of Neurosurgery, Showa Medical University School of Medicine, Tokyo, JPN; 2 Department of Neurosurgery, Showa Medical University Koto Toyosu Hospital, Tokyo, JPN; 3 Department of Radiology, Showa Medical University School of Medicine, Tokyo, JPN

**Keywords:** cavernous sinus lesions, differential diagnosis, endodermal cyst, epidermoid cyst, schwannoma, trigeminal nerve

## Abstract

Endodermal cysts of the cavernous sinus are extremely rare. We report the case of a 36-year-old man who presented with spontaneous pain in the ophthalmic (V1) and maxillary (V2) divisions of the trigeminal nerve. Based on the preoperative imaging findings, a cystic trigeminal schwannoma was suspected. The tumor was removed via an extradural middle fossa transcavernous approach, and histological examination confirmed the diagnosis of an endodermal cyst. Endodermal cysts should be considered in the differential diagnosis of cavernous sinus tumors.

## Introduction

Endodermal cysts, also known as neurenteric or enterogenous cysts, are rare congenital lesions of the central nervous system that arise from endodermal tissue displaced during embryogenesis. They represent only a small fraction of intracranial tumors (approximately 0.2-0.3%) [1,2], and most commonly occur in the spinal canal or posterior fossa. Supratentorial endodermal cysts are uncommon, and cavernous sinus involvement is particularly rare [3,4]. There are several theories about the mechanism of development of endodermal cysts that occur in unusual locations, such as anomalous endodermal cell migration [3,5-8]. To date, only two cases of cavernous sinus endodermal cysts have been documented, one in a child and one in an elderly person [9,10]. These lesions often lack pathognomonic imaging features and can be misdiagnosed as more frequently encountered cystic tumors, such as trigeminal schwannomas or epidermoid cysts [9,11]. Herein, we report an extremely rare case in a young adult with a right cavernous sinus endodermal cyst that was preoperatively presumed to be a cystic trigeminal schwannoma based on imaging features. The tumor was removed via an extradural middle fossa transcavernous approach, and histological examination confirmed the presence of an endodermal cyst. In this report, we describe this case, review the diagnostic challenges, discuss the salient radiological characteristics, and address the possible differential diagnoses and surgical considerations.

## Case presentation

**Figure 1:**
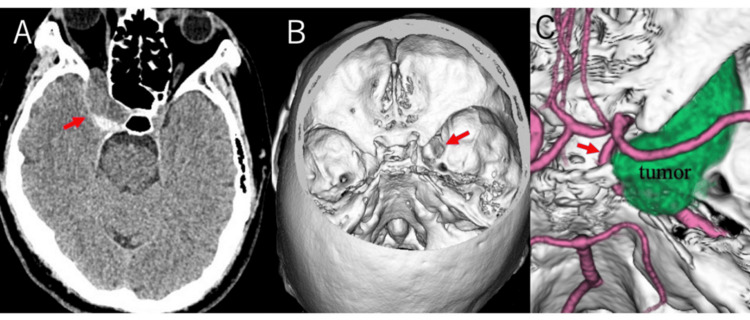
Preoperative images. A: Head computed tomography (CT) showing a cystic lesion within the right cavernous sinus (red arrow). B: The tumor enlarged the superior orbital fissure (red arrow). C: The tumor was adherent to the lateral wall of the cavernous segment of the internal carotid artery (red arrow).

**Figure 2:**
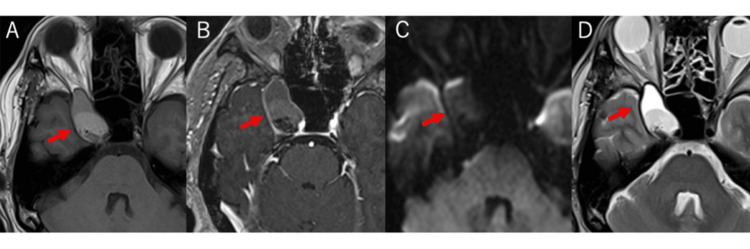
MRI images. A: T1-weighted image; B: Gadolinium-diethylenetriaminepentaacetate (Gd-DTPA) image; C: Diffusion-weighted image; D: T2-weighted image. MRI showed a multiloculated cyst containing fluid of mixed intensity, with no solid contrast-enhancing component.

A 36-year-old man presented with spontaneous sharp and shooting pain in the ophthalmic (V1) and maxillary (V2) divisions of the right trigeminal nerve. Neurological examination, including the extraocular muscle paresis, was unremarkable apart from the trigeminal pain distribution. Computed tomography (CT) of the head revealed a cystic lesion within the right cavernous sinus, markedly expanding the sinus and extending into the superior orbital fissure (Figure 1). The cyst showed predominantly low density with a dorsally high-density focus suggestive of hemorrhage. Magnetic resonance imaging (MRI) demonstrated a multiloculated cyst containing fluid of mixed intensity, with no solid contrast-enhancing component (Figure 2). Diffusion restriction was absent, arguing against an epidermoid cyst. Cerebral angiography revealed no abnormal vascularity or shunting. Based on these findings, a preoperative diagnosis of cystic trigeminal schwannoma was made, given the rarity of endodermal cysts in this location.

**Figure 3:**
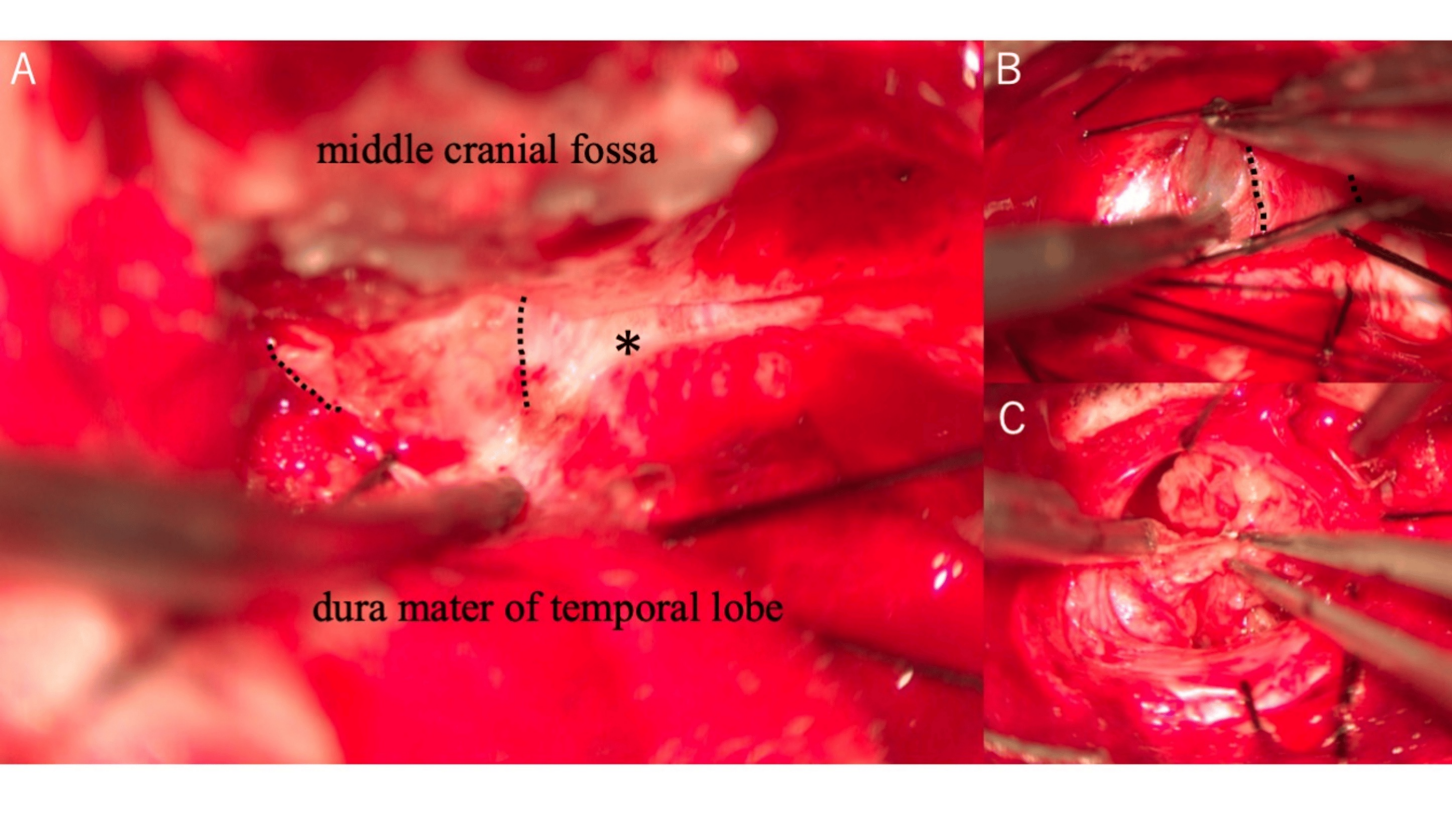
Intraoperative findings. A: The tumor capsule (asterisk) was opened posterior to V1 (dotted lines), revealing hemorrhagic fluid under pressure. B: Decompression facilitates visualization of the cranial nerves, including the thinned V2 (dotted lines). C: A solid component was also identified and resected piecemeal.

**Figure 4:**
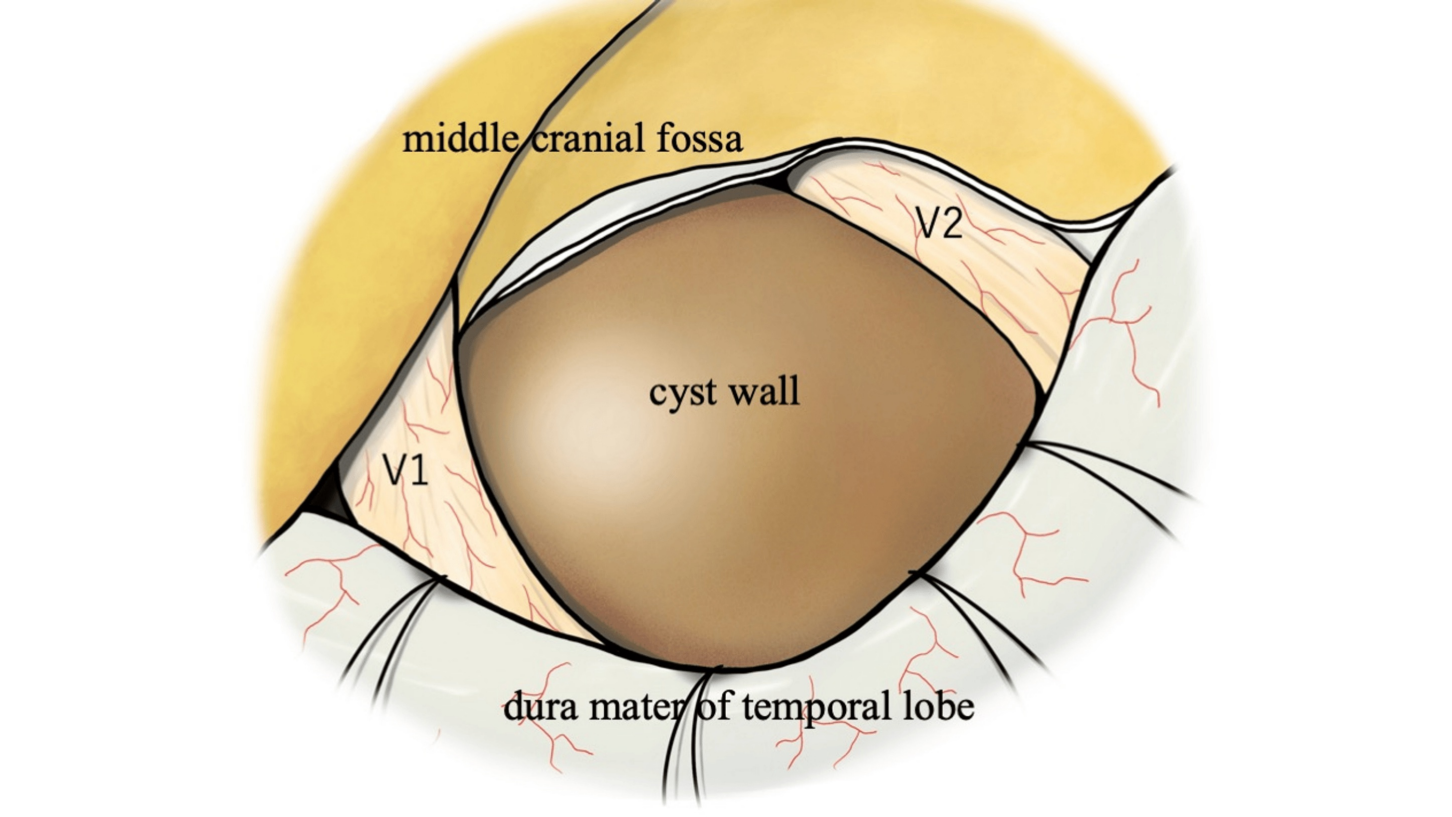
Schematic illustration of the operative field. We selected the approach to the cavernous sinus via the V1/V2 space (anteromedial triangle), where the cyst wall could be confirmed intraoperatively. Image credit: Dr. Ryo Irie. Permission to use the image has been obtained.

The patient underwent a right frontotemporal craniotomy using an extradural middle fossa transcavernous approach (Figures 3-4). After raising the bone flap, an interdural dissection was performed along the lateral wall of the cavernous sinus. The tumor capsule was opened posterior to V1, revealing hemorrhagic fluid under pressure. Decompression facilitated visualization of the cranial nerves, including a thinned V2. The solid component was identified and resected piecemeal. The lesion was firmly adherent to the lateral wall of the cavernous segment of the internal carotid artery, and a small portion of the capsule was intentionally left behind to avoid arterial injury.

**Figure 5:**
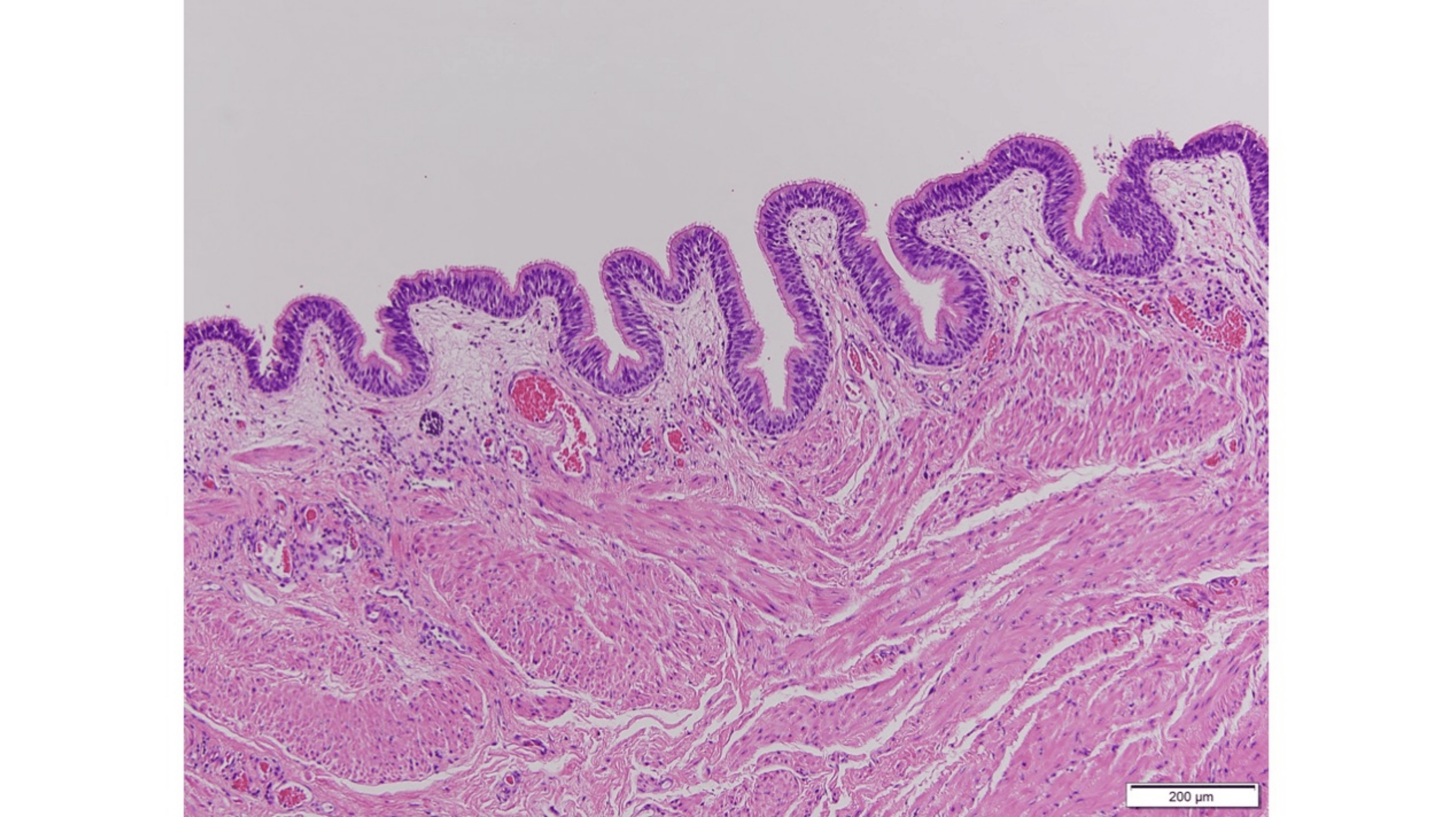
Pathological findings. Pathological examination revealed respiratory-type ciliated columnar epithelium with squamous metaplasia, mucus-secreting cells, and smooth muscle in the stroma, confirming an endodermal cyst.

Pathological examination revealed respiratory-type ciliated columnar epithelium with squamous metaplasia, mucus-secreting cells, and smooth muscle in the stroma, confirming the diagnosis of an endodermal cyst (Figure 5).

**Figure 6:**
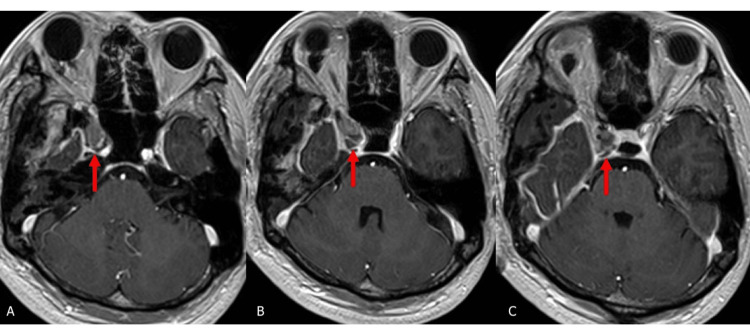
Postoperative MRI images. A-C: Gadolinium-diethylenetriaminepentaacetate (Gd-DTPA)-enhanced image showing postoperative status (red arrow).

Postoperatively, the patient’s trigeminal neuralgia resolved completely, and he remained neurologically intact at nine months (Figure 6).

## Discussion

Intracranial endodermal cysts are exceedingly rare, particularly in the cavernous sinus [9]. Our patient’s lesion presented with mixed cystic and hemorrhagic features, initially favoring the diagnosis of a cystic trigeminal schwannoma. This misdiagnosis is understandable, as cavernous sinus lesions more commonly include schwannomas, meningiomas, or inflammatory entities [10,11]. However, the absence of a solid enhancing component and the presence of mixed signal intensities in different parts of the cyst, without diffusion restriction, pointed to an alternative diagnosis. Endodermal (neurenteric) cysts can exhibit variable signals depending on the protein content or hemorrhage, often precluding a definitive diagnosis until histopathological confirmation [1,2,9].

Although a few cases of cavernous sinus endodermal cysts have been reported, they highlight shared diagnostic challenges (Table 1). Elshamy et al. described a cyst initially suspected to be a craniopharyngioma or an epidermoid [9]. Similarly, our case underscores the difficulty of distinguishing endodermal cysts from the more frequent cystic tumors based on imaging alone. Endodermal cysts often lack diffusion restriction (which would point to an epidermoid) and may not show the robust enhancement typical of schwannomas.

**Table 1:** Clinical characteristics of cavernous sinus endodermal cysts: a literature review. M: male; F: female; ICA: internal carotid artery; MRI: magnetic resonance imaging; V1: ophthalmic division of trigeminal nerve; V2: maxillary division of trigeminal nerve; V3: mandibular division of trigeminal nerve

Author (Year)	Age/Sex	Presenting Symptoms	Cyst Location	Preoperative Diagnosis	Surgical Approach	Extent of Resection
de Oliveira et al. (2005) [10]	2/F	Acute onset of third cranial nerve palsy (ophthalmoplegia)	Cavernous sinus	Not specified prior to surgery	Craniotomy with cyst aspiration and partial resection	Partial resection (could not be completely excised due to adherence to vital structures)
Elshamy et al. (2023) [9]	75/F	Gradually worsening headaches and left facial pain (V2-V3 distribution) over 6 months	Left cavernous sinus with encasement of cavernous ICA; widening of left foramen ovale and superior orbital fissure	Cystic trigeminal schwannoma, craniopharyngioma, or dermoid/epidermoid tumor	Extradural transcavernous approach	Gross total resection (confirmed) on postoperative MRI
Present case	36/M	Trigeminal (V1-V2) distribution of facial pain	Right cavernous sinus with extension into a superior orbital fissure	Cystic trigeminal schwannoma	Extradural transcavernous approach	Near-total resection (small capsule portion adherent to cavernous ICA left in situ)

Complete surgical resection is ideal to prevent recurrence. However, in the cavernous sinus, critical neurovascular structures often require subtotal removal when the cyst capsule is firmly adherent to the internal carotid artery [10]. In such cases, leaving a capsule remnant is reasonable to avoid iatrogenic injury, with close long-term follow-up imaging to detect regrowth. Our patient had a good course at nine months, which is consistent with other reports indicating that partial resection can be sufficient in the short term, although vigilant monitoring is necessary [9,10]. de Oliveira et al. reported a two-year-old girl with an endodermal cyst in the cavernous sinus who was reoperated following a recurrence after a three-year follow-up [10]. Moreover, although the malignant transformation of an endodermal cyst is extremely rare, documented cases of adenocarcinoma arising from recurrent neurenteric cysts underscore the importance of thorough excision and ongoing surveillance [12].

In summary, this case highlights the importance of considering endodermal cysts in the differential diagnosis of atypical cystic and partially hemorrhagic lesions of the cavernous sinus. Distinguishing it from schwannomas or epidermoid cysts may be challenging, but certain radiological features, such as mixed signal intensity, absence of a solid enhancing component, and absence of diffusion restriction, should raise suspicion. Surgical exploration and histopathological examination are crucial for a definitive diagnosis.

## Conclusions

This is a single case of a rare pathology, which limits broader generalization. Nevertheless, it provides valuable insights into cavernous sinus endodermal cysts and underscores the importance of including them in the differential diagnosis of cystic lesions in the cavernous sinus region.
